# Supplementary figures and images for: CD4+ but not CD8+ T cells are required for protection against severe guinea pig cytomegalovirus infections
Source: PLoS Pathog. 2024 Nov 4;20(11):e1012515. doi: 10.1371/journal.ppat.1012515 (PMC11563410; doi:10.1371/journal.ppat.1012515)

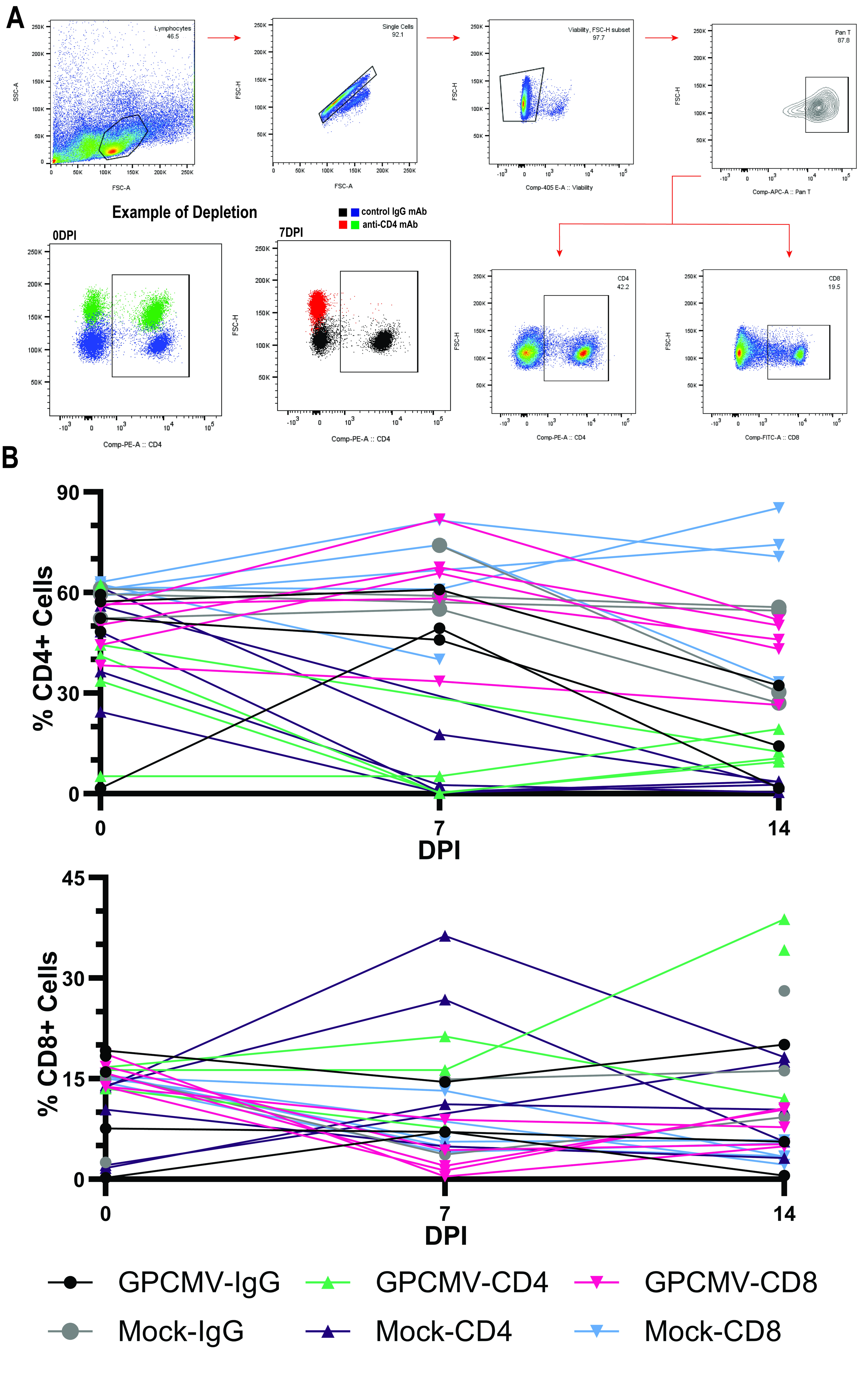

Supplement: S1 Fig — Flow cytometry was used to quantify the abundance of CD4+ and CD8+ cells among CD45+ PBMCs or splenocytes. (A) Gating strategy used for this analysis and representative flow plots showing the effects of rat IgG and α-CD4 on PBMCs at 0 and 7 days post-treatment. (B) The abundance of circulating CD4+ or CD8+ cells in individual pregnant guinea pigs that were antibody treated and either mock- or GPCMV-infected. (TIF) [file ppat.1012515.s001.tif]
