# Supplementary material for: CD4+ but not CD8+ T cells are required for protection against severe guinea pig cytomegalovirus infections
Source: PLoS Pathog. 2024 Nov 4;20(11):e1012515. doi: 10.1371/journal.ppat.1012515 (PMC11563410; doi:10.1371/journal.ppat.1012515)
Supplement: S2 Table — (PDF) [file ppat.1012515.s003.pdf]

S2 Table. Key Resource Table.		
**REAGENT or RESOURCE**	**SOURCE**	**IDENTIFIER**
**Chemicals, Peptides, and Recombinant Proteins**		
Dulbecco's Modified Eagle Medium (DMEM)	Gibco	11995-065
Roswell Park Memorial Institute 1640 (RPMI 1640)	Gibco	11875093
Fetal Bovine Serum (FBS)	Gibco	10082147
Penicillin Streptomycin (Pen-Strep)	Gibco	15140-122
MEM Non Essential Amino Acids	Gibco	11140-050
Dulbecco's Phosphate Buffer Saline (DPBS)	Gibco	14190-144
CELLine 1000 Bioreactor	Wheaton	WCL1000-1
Gibco 0.05% Trypsin-EDTA	Thermo Fisher Scientific	25300054
Dimethyl Sulfoxide (DMSO)	Sigma Aldrich	D2650
Trypan Blue Solution, 0.4%	Gibco	15250061
Bovine Serum Albumin	Thermo Fisher Scientific	BP9703
Sodium Azide	Sigma Aldrich	S2002
Shandon Formal-Fixx	Epredia	9990914
HiTrap MabSelect PrismA protein A column	Cytiva	17549851
Polyclonal Rat IgG	MP Biomedical	MFCD00164131
α-guinea pig CD4 IgG2b [Clone: H155]	Dr. Gregg Milligan	N/A
α-guinea pig CD8 mIgG2a [B607]	Dr. Gregg Milligan	N/A
α-guinea pig CD8 mIgG2b [Clone: 4F11]	Dr. Gregg Milligan	N/A
Rabbit Complement	Bio-Rad	C12CC
eBiosciences RBC Lysis Buffer	Thermo Fisher Scientific	00430054
Accutase TM	StemCell Technologies	07920
ddPCR Supermix for Probes (No dUTP)	Bio-Rad	1863024
Superfrost Plus slides	ThermoFisher	1255015
K2EDTA Blood Tubes	McKesson	229615
**Critical Commercial Assays**		
Progesterone ELISA	DRG	EIA-1561
RNAscope 2.5 HD Reagent Kit-RED	ACD	322452
RNAscope Probe - V-CavHV-2-*gp3*	ACD	322360-USM
DNEasy Blood and Tissue DNA Extraction Kit	Qiagen	69504

**Stains used for Flow Cytometry**		
Purified rat anti-mouse CD16/CD32 (Mouse BD Fc block) [Clone 2.4G2]	BD Biosciences	553142<br>RRID: AB_394656
RPE-conjugated mouse α-guinea pig CD4 [Clone: CT7]	Bio-Rad	MCA749PE<br>RRID: AB_609596
FITC-conjugated mouse α-guinea pig CD8 [Clone: CT6]	Bio-Rad	MCA752F<br>RRID: AB_321399
APC-conjugated mouse α-guinea pig T Lymphocytes [Clone: CT5]	Bio-Rad (discontinued in 2021)	MCA751APC<br>RRID: AB_567211
AF-405 conjugated mouse α-Guinea Pig CD45 [Clone: IH-1]	Bio-Techne	NB100-65362AF405<br>RRID: AB_960395
Ghost Dye Violet 510	Tonbo Biosciences	13-0870
PE-Cy7 conjugated mouse α-human CD14 [clone: M5E2]	Bio-Legend	301814<br>RRID: AB_389353
BV605 conjugated mouse α-human CD56 [clone: MY31]	BD Biosciences	742659<br>RRID: AB_2740950
**Experimental Models: Cell Lines**		
JH4 Cells	ATCC	CCL-158
H155 IgG2b (rat α-CD4, 4E5)	Gregg Milligan	N/A
B607 IgG2a (mouse α-CD8, 4F11)	Gregg Milligan	N/A
B607 IgG2a (mouse α-CD8, 2x clone)	Gregg Milligan	N/A
**Experimental Models: Organisms/Strains**		
Guinea Pig: Strain 13	USAMRIID	N/A
Guinea Pig: Strain 2	Dr. Mark Schleiss	N/A
**Oligonucleotides**		
GP54 Forward Primer	5'-ACGAACACAAGGACGATCTC-3'	
GP54 Reverse Primer	5'-CTTGATACAATACCGCCCGA-3'	
GP54 Probe	56-FAM/TCCCACCGA/ZEN/CTTTTCTCGGC/3IABkFQ	
GP Actb Forward Primer	5'-GAAGCCCAGAGCAAAAGAGGTA-3'	
GP Actb Reverse Primer	5'-CCATGTCGTCCCAGTTGGTAA-3'	
GP Actb Probe	5HEX/CTGACCCTG/ZEN/AAATACCCCATTGAGCAC/3IABkFQ	
GP Tspy2-like Forward Primer	5'-CTATCGTGAAGGAGTTTCTCTGG-3'	
GP Tspy2-like Reverse Primer	5'-GTAAACCAGTTGAAGAAGTTCGG -3'	
GP Tspy2-like Probe	5HEX/ATTCAGTGG/ZEN/CGTGCCAGTTACAGA/3IABkFQ	
**Software and Algorithms**		
BD FACSDiva	BD Biosciences	V8.0.1
FlowJo	https://www.flowjo.com/	Build v14.1
QuantaSoft Analysis Pro	BioRad	V1.4
QuPath	https://qupath.github.io/	Version 0.5.1
R	https://www.r-project.org/	Version 4.2.2
